# Functionalized AgFeO_2_ Particles as Fluorescent Platforms for the Detection of *Salmonella* spp.

**DOI:** 10.1021/acsomega.5c07267

**Published:** 2026-03-07

**Authors:** Lizeth C. Mojica-Sánchez, Gabriela P. Ratkovski, Bruna G. Maciel, Winnie Q. Brandão, Romário J. da Silva, Celso P. de Melo

**Affiliations:** † Departamento de Física, 28116 Universidade Federal de Pernambuco, 50670-901 Recife, Pernambuco, Brazil; ‡ Pós-Graduação em Ciência de Materiais, Universidade Federal de Pernambuco, 50670-901 Recife, Pernambuco, Brazil

## Abstract

Rapid and accurate pathogen detection remains a major challenge in public health, particularly in food safety and biomedical diagnostics. Here, we present a highly sensitive biosensing platform based on silver ferrite (AgFeO_2_) nanoparticles functionalized with 3-mercaptopropionic acid (3-MPA) and single-stranded DNA (ssDNA). The sensor enables ultrafast detection, with signal saturation reached in under 10 min, and achieves an analytical limit of detection (LoD) of 73 pmol L^–1^ for Salmonella spp. DNA sequences. The platform also exhibits excellent selectivity, clearly distinguishing fully complementary targets from sequences containing base mismatches. Its versatility is further enhanced by the incorporation of SYBR Green I dye, enabling dual fluorescence-based detection and on–off sensing of double-stranded DNA (dsDNA). By combining high sensitivity, selectivity, and operational simplicity, the AgFeO_2_@MPA/ssDNA biosensor demonstrates strong potential for applications in biotechnology, diagnostics, and food safety, with performance comparable to state-of-the-art biosensing technologies.

## Graphical abstract

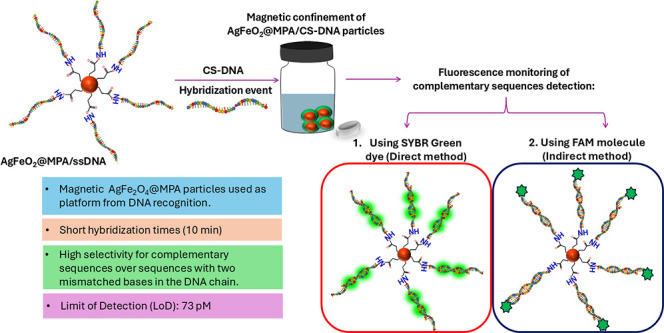

## 1 Introduction

Globally, nearly 10% of the population is affected by foodborne diseases each year.^1^ According to the World Health Organization (WHO), more than 200 types of pathogenic contaminants can be found in food at any stage of production, distribution, or consumption, causing approximately 420,000 deaths annually. Children under five years of age, who have underdeveloped immune systems and often lack access to safe food and water, account for a disproportionate 30% of these deaths.^2–4^

Contamination of food and beverages with harmful bacteria, viruses, parasites, and toxins can result from environmental pollution of water, soil, or air, as well as from improper food storage and processing.^5^ The effective detection of pathogens is a major public health issue that requires rapid, affordable, and practical solutions.^6^

Several methods can be used to detect the presence of *Salmonella* spp., including enzyme-linked immunosorbent assays (ELISAs),^7^ bacterial culture techniques,^8^ polymerase chain reaction methods,^9,10^ and, more recently, biosensor platforms.^11,12^ However, the procedures currently available for *Salmonella* detection are often labor-intensive and time-consuming, and they frequently lack adequate specificity and sensitivity, with suboptimal detection limits.^13^ In this context, biosensor technology emerges as a promising alternative to conventional techniques for identifying this pathogen, offering not only higher sensitivity but also faster operational times.^14–16^

Biosensors are analytical devices that consist of a recognition element coupled to a transducer system, capable of converting a biological event into a detectable signal.^17–19^ Literature provides various examples of methods for signal transduction using different materials that function as effective sensors. Among these, special mention should be made of transducers that use electrochemically active materials.^16^ Other notable examples include transducers that measure surface plasmon resonance using metallic nanomaterials,^20,21^ and fluorescence sensors that exploit the quenching of fluorophore-labeled DNA probes attached to gold nanoparticles.^22,23^

The use of nanomaterials enhances the sensitivity of biosensors, reduces detection time, and simplifies the handling process.^24–26^ Consequently, these nanomaterials have become integral to the design of in vitro diagnostic platforms, owing to their high surface-to-volume ratio, tunable surface chemistry, and selective interactions with biomolecules. Metal oxide nanostructures and hybrid nanoparticles have been widely employed to enhance DNA biosensor performance by enabling efficient probe immobilization, improving hybridization kinetics, and amplifying signal transduction. Their compatibility with diverse detection modalities—optical, electrochemical, and magnetic—further supports the development of portable, low-cost, and highly specific diagnostic tools for clinical, environmental, and food safety monitoring.

Specifically, magnetic nanoparticles (MNPs) have garnered significant attention because of their unique physicochemical properties—optical, electronic, and catalytic—arising from quantum confinement and surface effects at the nanoscale.^27^ In this study, we investigated the magnetic properties of MNPs to assess their versatility in functionalization with both organic and inorganic species.^28^ After interacting with biological complexes, MNPs can be readily separated using an external magnetic field, facilitating their application. The capacity to chemically functionalize MNPs—such as by anchoring molecules or activating specific sites for the covalent attachment of biomolecules—enhances their adaptability in biomedical assays.^29–32^ Although the covalent functionalization of MNPs with biomolecules involves multiple chemical conjugation steps, it enables the development of sensors that are both selective and robust.^33^ In this study, using the detection of *Salmonella* spp. as a model system, we introduce functionalized silver–iron oxide nanoparticles (AgFeO_2_ NPs) as a promising platform for recognizing single-stranded DNA (ssDNA) fragments of pathogens. To enable covalent attachment of amino-modified ssDNA via EDC coupling, we first introduced bifunctional organic ligands onto the silver–iron oxide nanoparticles (steps 1 and 2 in Figure 1). The resulting ssDNA-functionalized AgFeO_2_ nanoparticles are then exposed to 6-carboxyfluorescein-labeled complementary ssDNA (FAM-DNA), allowing hybridization to be monitored through changes in fluorescence intensity (step 4A, Figure 1).

**Figure 1:**
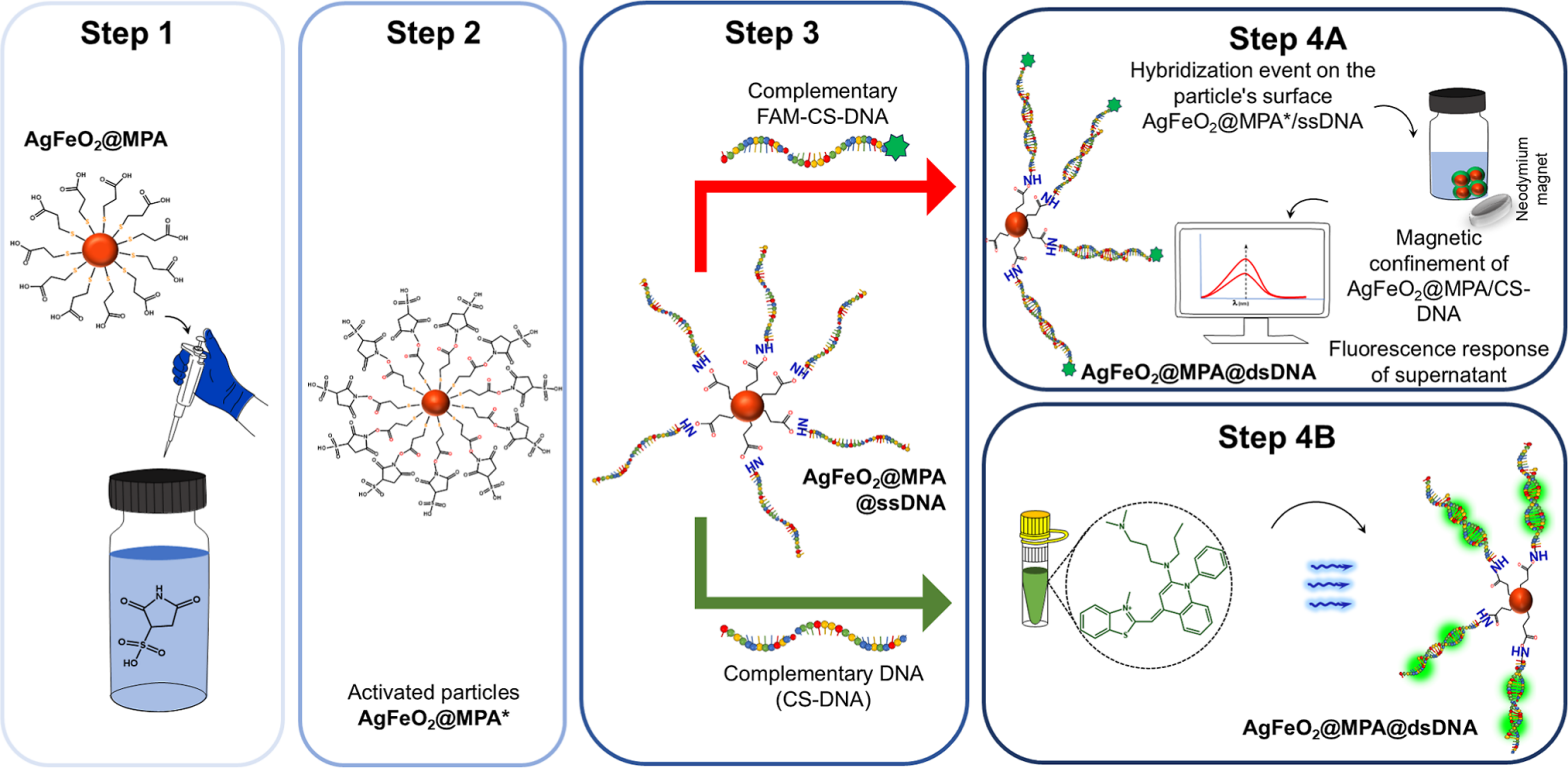
Schematic representation of the mechanism employing silver–iron oxide (AgFeO_2_) nanoparticles as platforms for recognizing specific DNA sequences. Steps 1–2 show the activation of COOH groups using EDC-NHS coupling, in step 3, the hybridization process is shown, and step 4a shows the detection using a FAM-labeled complementary DNA strand, where fluorescence change serves as the analytical signal; and step 4b shows the detection using SYBR Green I (SG) dye to identify double-stranded DNA hybridization.

As an alternative recognition method, we used the SYBR Green I (SG) fluorophore to detect the system’s response through fluorescence upon the formation of double-stranded DNA (dsDNA), indicating a specific hybridization event on the proposed platform. In this approach, we used the complementary DNA strand without the FAM-labeled probe. Due to its ability to increase brightness by more than 1000-fold upon binding to dsDNA, SG dye is widely employed in various DNA detection and analysis techniques.^34^ A schematic representation of the recognition mechanism is shown in step 4B in Figure 1.

## 2 Experimental Section

### 2.1 Materials

The reagents ferric nitrate nonahydrate (Fe­(NO_3_)_3_·9H_2_O) and silver nitrate (AgNO_3_), used as metal precursors for nanoparticle synthesis, as well as 2-(*N*-morpholino)­ethanesulfonic acid (MES), employed as a buffering agent, and the ligands 3-mercaptopropionic acid (3-MPA) and 11-mercaptoundecanoic acid (11-MUA), tris­(hydroxymethyl)­aminomethane (Tris), and bovine serum albumin (BSA) protein, were all purchased from Sigma-Aldrich (USA). Ammonium hydroxide (NH_4_OH) was obtained from Dinâmica (Brazil), sodium chloride (NaCl) from Química Moderna (Brazil), and sodium citrate tribasic dihydrate (Na_3_C_6_H_5_O_7_·2H_2_O) and sodium phosphate monobasic (NaH_2_PO_4_) from Nuclear (Brazil). *N*-(3-(dimethylamino)­propyl)-*N*′-ethylcarbodiimide hydrochloride (EDC) and *N*-hydroxysulfosuccinimide (sulfo-NHS) were purchased from Thermo Fisher Scientific (USA). All the above reagents, of analytical grade, were used without further purification. Table 1 lists the oligonucleotide sequences used in this study, which were purchased in purified form from Thermo Fisher Scientific (USA). All NH-ssDNA sequences used for immobilization were designed to target a specific region of the *Salmonella* spp. genome (strain FSIS1607447, GenBank accession no. CP082473; chromosome positions 1,002,571–1,002,596, rpoS gene), as verified by BLAST analysis (National Center for Biotechnology Information).

**Table 1:** Oligonucleotide Sequences are Used in This Study

name	oligonucleotide sequences	symbol
NH-ssDNA-Salmonella spp immobilization	5′NH_2_-GTG AAA TTA TCG CCA CGT TCG GGC AA-3′	NH-ssDNA
poly A-NH-ssDNA poly A-immobilization	5′NH_2_-AAA AAA AAA AAA GTG AAA TTA TCG CCA CGT TCG GGC AA-3′	Poly A-ssDNA
complementary target ssDNA-FAM	5′-FAM-TTG CCC GAA CGT GGC GAT AAT TTC AC-3′	CS-DNA-FAM
complementary target ssDNA without FAM	5′-TTG CCC GAA CGT GGC GAT AAT TTC AC-3′	CS-DNA
noncomplementary target	5′-FAM-GCC TGG ATA GTA ACG TAC ATG-3′	NCS-DNA
double-based mismatched target ssDNA (middle)	5′-FAM-TTG CCC GAA CGA GCC GAT AAT TTC AC-3′	T2M
double-based mismatched target ssDNA (beginning)	5′-FAM-TTG CCC GAA CGT GGC GAT AAT TTG AG-3′	T2I

We selected poly-A sequences to study the immobilization procedure with a spacer. The sequences designated as CS are the specific complementary strands for the immobilized sequence, while those labeled NCS are the noncomplementary strands. The sequences named T2M and T2I contain two mismatched bases in the complementary sequence. Ultrahigh-purity water (Millipore, USA) was used in all bioconjugation protocol experiments and nanoparticle syntheses.

### 2.2 Synthesis of AgFeO_2_ Nanoparticles

AgFeO_2_ nanoparticles were synthesized using a coprecipitation method in an alkaline medium, with Fe­(NO_3_)_3_·9H_2_O and AgNO_3_ as metal precursors, and NH_4_OH as the precipitating agent.^32^ The synthesis was conducted in a reflux system maintained at a constant temperature of 100 °C. First, 25 mL of a 1 mol·L^–1^ Fe­(NO_3_)_3_·9H_2_O solution and 25 mL of a 1 mol·L^–1^ AgNO_3_ solution were mixed in a 250 mL round-bottom flask. After 10 min, 125 mL of a 50% v/v NH_4_OH solution was added under vigorous stirring. The solution was then kept at 100 °C for 2 h. After this period, the precipitate was separated using a magnet. The collected material was washed several times with deionized water and subsequently dried in an oven at 60 °C for 12 h.

The synthesized nanoparticles were subjected to thermal treatment to remove any residual organic species. For this purpose, the AgFeO_2_ NPs were placed in an electric furnace at 200 °C for 24 h, then collected and stored for subsequent analyses (Figure SI1a, in the Supporting Information).

### 2.3 Functionalization and Bioconjugation Protocol

In this study, we used oligonucleotide sequences with terminal amine groups, as listed in Table 1. A preliminary step for bioconjugating *Salmonella* spp. ssDNA sequences onto AgFeO_2_ NPs involved the chemical incorporation of a bifunctional ligand (i.e., molecules containing both thiol and carboxylic groups). To this end, we examined the effect of bifunctional molecules with different chain lengths on the efficiency of NH_2_-ssDNA immobilization on AgFeO_2_ NPs.

#### 2.3.1 MPA-Modification of AgFeO_2_ NPs

We compared 3-MPA with the longer-chain 11-MUA as bifunctional ligands for modifying the silver–iron oxide nanoparticles, to assess how ligand chain length influences nonspecific ssDNA adsorption. For the functionalization process, 100 mg of AgFeO_2_ NPs and 25 mL of a 10 mmol·L^–1^ 3-MPA (or 11-MUA) solution were placed in a 50 mL round-bottom flask. The mixture was stirred vigorously for 24 h, and the final product was dried at 60 °C, as shown in Figure SI1b.

#### 2.3.2 Bioconjugation Procedures: Immobilization of ssDNA on AgFeO_2_@MPA Particles

The bioconjugation of *Salmonella* spp. ssDNA involved coupling the primary amines (−NH_2_) of the modified oligonucleotide with the carboxylic groups (−COOH) of 3-MPA (or 11-MUA) through carbodiimide-mediated reactions, resulting in the formation of covalent amide bonds.^35^ This process is performed in sequential steps. The first step involves activating the –COOH groups using EDC/sulfo-NHS coupling chemistry, with a 0.1 mol·L^–1^ MES solution at pH 4.7 as the activation buffer.^36^ For this, 500 μL of a 2 mmol·L^–1^ EDC solution and 500 μL of a 5 mmol·L^–1^ sulfo-NHS solution, both dissolved in the MES activation buffer, were added to 1 mL of a 0.5 mg·mL^–1^ AgFeO_2_@MPA (or AgFeO_2_@MUA) solution. After gently shaking the activation mixture for 30 min, the activated particles (AgFeO_2_@MPA* or AgFeO_2_@MUA*) were magnetically separated and washed with a coupling buffer consisting of a 0.1 mol·L^–1^ MES solution at pH 7.4.^37,38^

The second step involved covalent coupling between the –COOH groups of AgFeO_2_@MPA* (or AgFeO_2_@MUA*) and the amine-modified ssDNA strands. For this step, 1 mL of a 40 nmol·L^–1^ NH-ssDNA solution was added to 0.5 mg of AgFeO_2_@MPA* particles, and the system was gently shaken for 2 h. Subsequently, the resulting AgFeO_2_@MPA/ssDNA complex particles, containing the incorporated single-stranded DNA chains, were washed with a 0.1 mmol·L^–1^ phosphate buffer solution to remove nonspecifically bound DNA molecules. The active site blocking step was carried out in step 3:1 mL of a 0.5 mol·L^–1^ BSA solution was added to block any remaining unreacted sites, and the mixture was kept at 5 °C for 16 h. Finally, the AgFeO_2_@MPA@NH-ssDNA (or AgFeO_2_@MUA@NH-ssDNA) complex particles were magnetically separated, washed with the coupling buffer, and stored for the subsequent hybridization procedure.^39–41^

### 2.4 Detection of CS-DNA-FAM Complementary Probes

#### 2.4.1 Hybridization Procedure

As an initial approach for detecting CS-DNA probes, we incubated the AgFeO_2_@MPA@NH-ssDNA (or AgFeO_2_@MUA@NH-ssDNA) nanoparticles with FAM-labeled CS-DNA. For this step, the complex particles were resuspended in 1 mL of a 5 nmol·L^–1^ CS-DNA-FAM solution in a 6× saline-sodium citrate (SSC) hybridization buffer. This buffer was prepared by diluting a 20× stock solution, which contains 3 mol·L^–1^ NaCl and 300 mmol·L^–1^ sodium citrate, resulting in a final 6× concentration of 0.9 mol·L^–1^ NaCl and 90 mmol·L^–1^ sodium citrate at pH 8.4. The hybridization reaction was allowed to proceed for 120 min under gentle shaking, after which the nanoparticles were magnetically separated.

#### 2.4.2 Sensing Performance: Fluorescence Measurements

In the fluorescence sensing assays, 0.5 mg of AgFeO_2_@MPA@NH-ssDNA (or AgFeO_2_@MUA@NH-ssDNA) particles were allowed to interact with 1 mL of the target CS-DNA-FAM, diluted in the hybridization buffer, under gentle shaking for 120 min. Target concentrations ranged from 0.05 to 10.0 nmol·L^–1^. Afterward, the hybridized particles were magnetically separated and washed twice with the hybridization buffer, and the fluorescence intensity of the supernatant was measured.

The amount of the CS-DNA effectively coupled with its complementary strand, forming the FAM-labeled AgFeO_2_@MPA@NH-dsDNA (or AgFeO_2_@MUA@NH-dsDNA) particles, was calculated by measuring the decrease in fluorescence intensity of the solution after these complex particles were removed from the medium.

### 2.5 Detection of Nonlabeled CS Probes Using SYBR-Green

The SYBR-Green dye exhibits intense fluorescence only when it interacts with double-stranded DNA. We utilized this property in an alternative approach to assess the effectiveness of the hybridization process with nanoparticles containing the nonlabeled complementary (CS) strands and the AgFeO_2_@MPA@NH-ssDNA particles.

In this procedure, we used label-free CS to form the AgFeO_2_@MPA@ssDNA complex particles, following the hybridization methodology. After washing the freshly prepared AgFeO_2_@MPA@dsDNA particles twice with the hybridization buffer and once with nuclease-free ultrapure water, 1 mL of a 1× diluted SG dye solution was added. The SG dye was diluted in deionized, filtered, autoclaved water that was tested for contaminants such as endonucleases, exonucleases, and RNases. (The stock solution of SG dye is a 10,000× concentrate in DMSO.) The mixture was gently shaken for 30 min.

Afterward, the fluorescence signal of the AgFeO_2_@MPA@dsDNA particles was measured using a Zeiss Axio Imager 2 fluorescence microscope equipped with an Axiocam 503c_s256 camera.

For all bioconjugation experiments, we used 0.5 mg of AgFeO_2_@MPA/ssDNA particles and 1 mL of either CS-DNA, NCS-DNA, T2M/T2I, or SG dye solution, with concentrations specified in each section.

## 3 Characterization Methods

The structure of the AgFeO_2_ nanoparticles was characterized by X-ray diffraction (XRD) using Cu Kα radiation (λ = 1.5406 Å) on a Rigaku SmartLab instrument (Japan). The corresponding FTIR spectra for AgFeO_2_ and AgFeO_2_@MPA were collected in the 4000–400 cm^–1^ range, using KBr pellets on an IRTracer-100 spectrophotometer (Shimadzu, Japan).

To measure the fluorescence response of the AgFeO_2_@MPA@NH-ssDNA complex and FAM-labeled CS probes, we used a FluoroLog-3 spectrofluorometer (Horiba, USA). The fluorescence spectra were collected in the 490 to 650 nm range, with an excitation wavelength of 480 nm. Hysteresis loop measurements were performed using a Vibrating Sample Magnetometer (VSM) EV7 (MicroSense, USA). To examine the morphology of the AgFeO_2_ particles, we used a MIRA 3 SEM microscope (TESCAN) in STEM mode. Finally, the –COOH concentration in the AgFeO_2_@MPA particles after functionalization was estimated via a colorimetric assay based on Toluidine Blue adsorption.^42^

## 4 Results and Discussion

### 4.1 Characterization of AgFeO_2_ NPs

After obtaining the XRD diffraction pattern of the AgFeO_2_ nanoparticles, we refined the structural data using the Rietveld method with Maud software. The corresponding results are shown in Figure 2.

**Figure 2:**
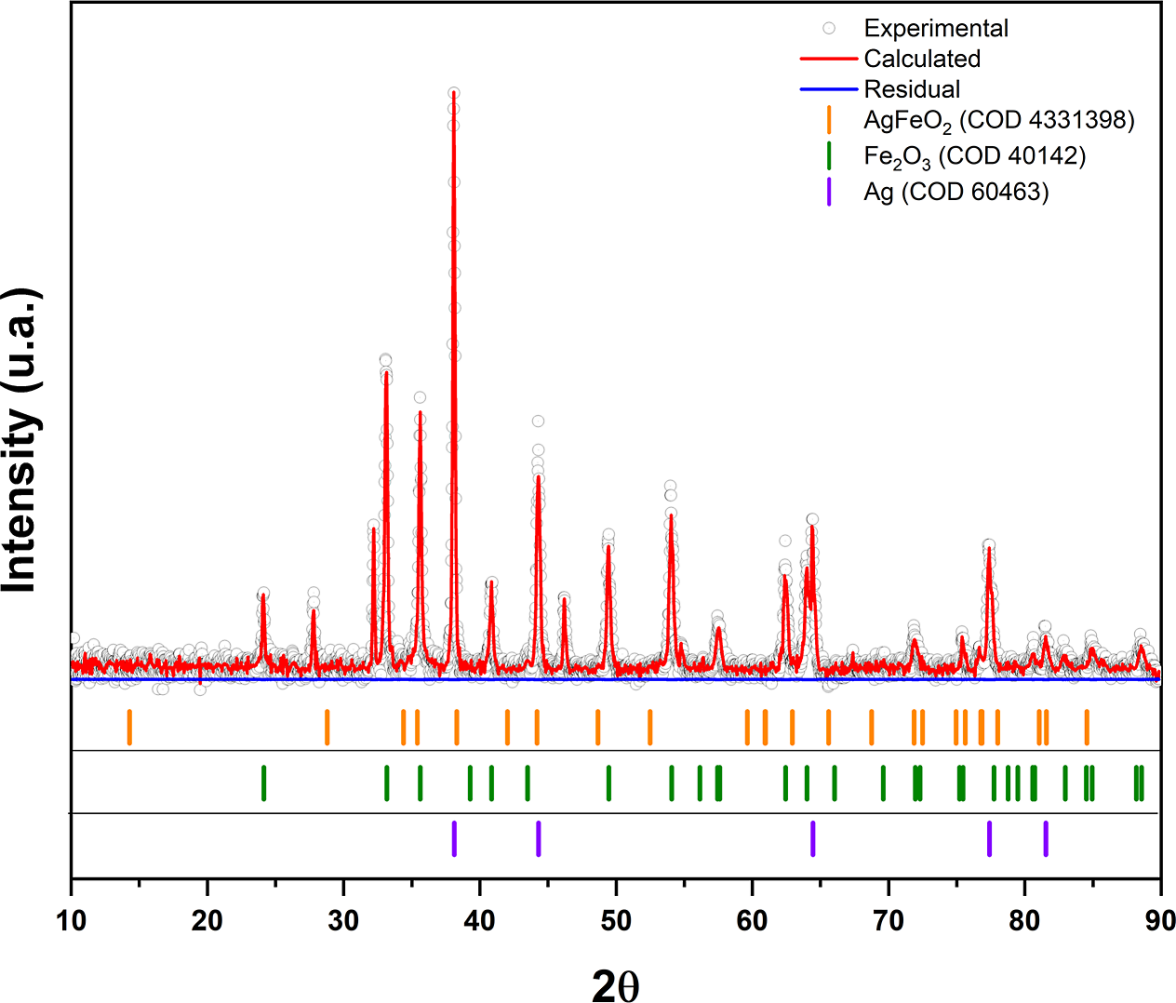
X-ray diffraction (XRD) patterns of AgFeO_2_ nanoparticles, showing experimental data, calculated fit, and residuals, with phase identifications for AgFeO_2_, Fe_2_O_3_, and Ag.

The peaks observed for the AgFeO_2_ phase correspond to a rhombohedral structure with space group *R*3̅*m* (COD number 4331398), and reflection planes (012), (021), (1013), and (027) at 2θ angles of 35.41°, 71.86°, 75.61°, and 81.57°, respectively. These reflection planes confirm the presence of a trigonal structure and validate the identification of the delafossite AgFeO_2_ particles.^43–46^ Additionally, peaks were observed at 2θ angles of 24.20°, 33.23°, 35.7°, and 40.95°, corresponding to the reflection planes (012), (104), (110), and (113) of the Fe_2_O_3_ phase with a trigonal structure and space group *R*3̅*c* (COD number 40142).^47,48^ Peaks at 2θ angles of 38.20°, 44.40°, 64.60°, 77.54°, and 81.75° correspond to the reflection planes (111), (200), (220), (311), and (222) of the cubic Ag phase with space group *Fm*3̅*m* (COD number 604630).^49,50^ In this context, the presence of byproduct phases such as Fe_2_O_3_ and Ag is attributed to the synthesis method and the molar ratios used during the process. However, these byproducts do not interfere with the bioconjugation protocols. It is important to note that during the bioconjugation and washing/purification steps, the nanoparticles were separated using a neodymium magnet, ensuring that only magnetically responsive particles were collected.

### 4.2 Characterization of MPA-Functionalized AgFeO_2_ NPs

#### 4.2.1 FTIR Spectra

In Figure SI2, we present the FTIR spectra collected in the 4000–400 cm^–1^ range for the pristine AgFeO_2_ particles (curve i), the functionalized AgFeO_2_@MPA particles (curve ii), and the 3-MPA ligand (curve iii). No organic species were found on the surface of the pristine particles (see curve i), and peaks A and B at 448 cm^–1^ and 581 cm^–1^ can be attributed to the stretching vibrations of silver–oxygen (Ag–O) and iron–oxygen (Fe–O) bonds, respectively.^51,52^ The peak at 1624 cm^–1^ is associated with out-of-plane and in-plane vibrations of absorbed water molecules (O–H), similar to the peak at 3303 cm^–1^.^52^ For the functionalized particles (curve ii), new peaks appear in the 2000–1000 cm^–1^ region, particularly peaks C (1618 cm^–1^) and D (1407 cm^–1^), which can be attributed to the asymmetric and symmetric vibrations of the carboxylate anion (COO^–^).^53,54^ Peaks A and B still correspond to the metal–oxygen bonds. In both curves ii and iii, peak E at 3443 cm^–1^ is attributed to O–H bonds, while peak F at 2900 cm^–1^ corresponds to the asymmetric and symmetric stretching of CH_3_ groups. For the 3-MPA ligand in curve iii, peak J at 1200 cm^–1^ corresponds to C=O vibrations and C–O stretching modes. Peak G at 2500 cm^–1^ can be attributed to the S–H bond, which disappears in the functionalized particles (curve ii), indicating an interaction between the −SH group and Ag^+^ particles, confirming that the thiol groups of the acid are attached to the AgFeO_2_ particles via sulfur–silver bonds.^55^ The intense peak H at 1700 cm^–1^ is attributed to the C=O bond of the carboxylic group. Overall, these results confirm that surface functionalization of the nanoparticles was successfully achieved.

#### 4.2.2 Determination of –COOH Concentration on AgFeO_2_@MPA Particles

The Toluidine Blue (TB) dye adsorption method, used to determine the COOH concentration on the surface of AgFeO_2_ nanoparticles, is based on the coordination of 1 mol of COOH with 1 mol of TB dye. This allows the amount of adsorbed TB dye to be directly correlated with the number of COOH groups present on the particle’s surface.^42,56^ The concentration of surface carboxyl (−COOH) groups on the AgFeO_2_@MPA nanoparticles was estimated as (96.21 ± 0.0034) mmol g^–1^ of AgFeO_2_. After functionalization, an increase in the stability of the particle dispersion was observed, which is advantageous for the subsequent steps in the construction of the platform.

#### 4.2.3 ζ-potential Measurements

Successful functionalization was further confirmed by ζ-potential measurements of pristine AgFeO_2_ and AgFeO_2_@MPA nanoparticles in aqueous solution at pH 7. The measured values, (+12.36 ± 6.38) mV for pristine AgFeO_2_ and (−7.54 ± 1.32) mV for AgFeO_2_@MPA, are consistent with the functionalization process involving carboxylic molecules, as carboxylate anions (COO^–^) are formed in aqueous dispersions.^57,58^ This confirms that the iron–silver particles obtained are effective substrates for surface linkage of other molecules via bioconjugation procedures.

To further investigate the morphology of the AgFeO_2_ particles, we used STEM mode, as shown in Figure SI3. As observed, the particles tend to form agglomerates due to magnetic interactions. Although the structure of individual particles cannot be clearly identified, the particles appear to have a roughly spherical morphology, though this has not been definitively confirmed.

The hysteresis curve at room temperature (Figure SI4) is characteristic of “soft ferromagnetic” behavior, consistent with the magnetization value (8.96 emu·g^–1^ at *M*_Hmax_ = 30 kOe) and the small coercivity field (Hc = 51.13 Oe), similar to values observed for silver–iron oxide prepared by the coprecipitation method.^59,60^

### 4.3 Biosensor Construction: Use of AgFeO_2_@MPA/ssDNA as Sensors for Specific Nucleotide Sequences

Our strategy was to achieve a robust attachment of ssDNA sequences to AgFeO_2_@MPA (or AgFeO_2_@MUA) particles in the initial step, through the formation of covalent bonds. The resulting AgFeO_2_@MPA/ssDNA complex particles could then be used as reliable platforms for sensing target DNA sequences of specific pathogens. In this study, we used single-stranded DNA sequences specific to *Salmonella* spp., modified with NH_2_ groups (NH_2_-ssDNA), as our model system.

To assess the effectiveness of the proposed platform, we exposed 0.5 mg of the prepared AgFeO_2_@MPA/ssDNA particles to 1 mL of a 5 nmol·L^–1^ solution of FAM-labeled complementary NH_2_-ssDNA in the second step. As a result, double-stranded DNA (dsDNA) chains were formed on the AgFeO2@MPA/ssDNA particles. Afterward, these AgFeO_2_@MPA/dsDNA particles were magnetically separated and washed with a 0.1 mol·L^–1^ phosphate solution to remove any ssDNA sequences that were not covalently attached. The corresponding decrease in fluorescence intensity of the drained solution was directly related to the amount of AgFeO_2_@MPA/dsDNA formed.

In the first step, the degree of immobilization was estimated from the fluorescence intensity according to *f*_Imm_ = 100 × (*I*_0_-*I*_SN_)/*I*_0_, where *I*_0_ and *I*_SN_ are the fluorescence intensities of the initial ssDNA solution and the supernatant collected after removal of the AgFeO_2_@MPA/ssDNA nanoparticles, respectively, during the washing process. As shown in Figure 3a, when using 1 mL of a 100 nmol·L^–1^ FAM-labeled NH_2_-ssDNA solution, a degree of immobilization of 66.7 ± 2.54% was observed (rectangle with diagonal hatching). During the washing process with phosphate solution, 56% (55.7 ± 2.41%) of the FAM-labeled NH_2_-ssDNA solution was released from the particles (rectangle with horizontal hatching). Therefore, a significant portion of the attached ssDNA sequences was bound through nonspecific interactions, such as hydrogen bonds. As a result, approximately 11% of the NH_2_-ssDNA (15 nM out of an initial 100 nM) successfully formed amide bonds with the AgFeO_2_@MPA nanoparticles. We also estimated the degree of immobilization when 1 mL of a 40 nmol·L^–1^ FAM-labeled NH_2_-ssDNA solution was used. In this case, 90.1 ± 1.07% of the ssDNA was immobilized (indicated by the rectangle with diagonal hatching), but 51.8 ± 3.07% was released after phosphate washing (indicated by the rectangle with horizontal hatching). Therefore, approximately 38% of the ssDNA (16 nM out of an initial 40 nM) was successfully attached to the AgFeO_2_@MPA nanoparticles. Because 1 mL of a 40 nmol L^–1^ FAM-labeled NH_2_-ssDNA solution was sufficient to saturate the active sites on the AgFeO_2_@MPA surface, this concentration was used for all subsequent hybridization and selectivity experiments. Using a lower initial DNA concentration substantially reduced molecular losses. Although the absolute amount of attached ssDNA (≈16 nM) was similar to that obtained at 100 nM (≈15 nM), immobilization efficiency was higher at 40 nM. Under these conditions, the available active sites captured and retained ssDNA more effectively, minimizing waste and maximizing surface coverage.

**Figure 3:**
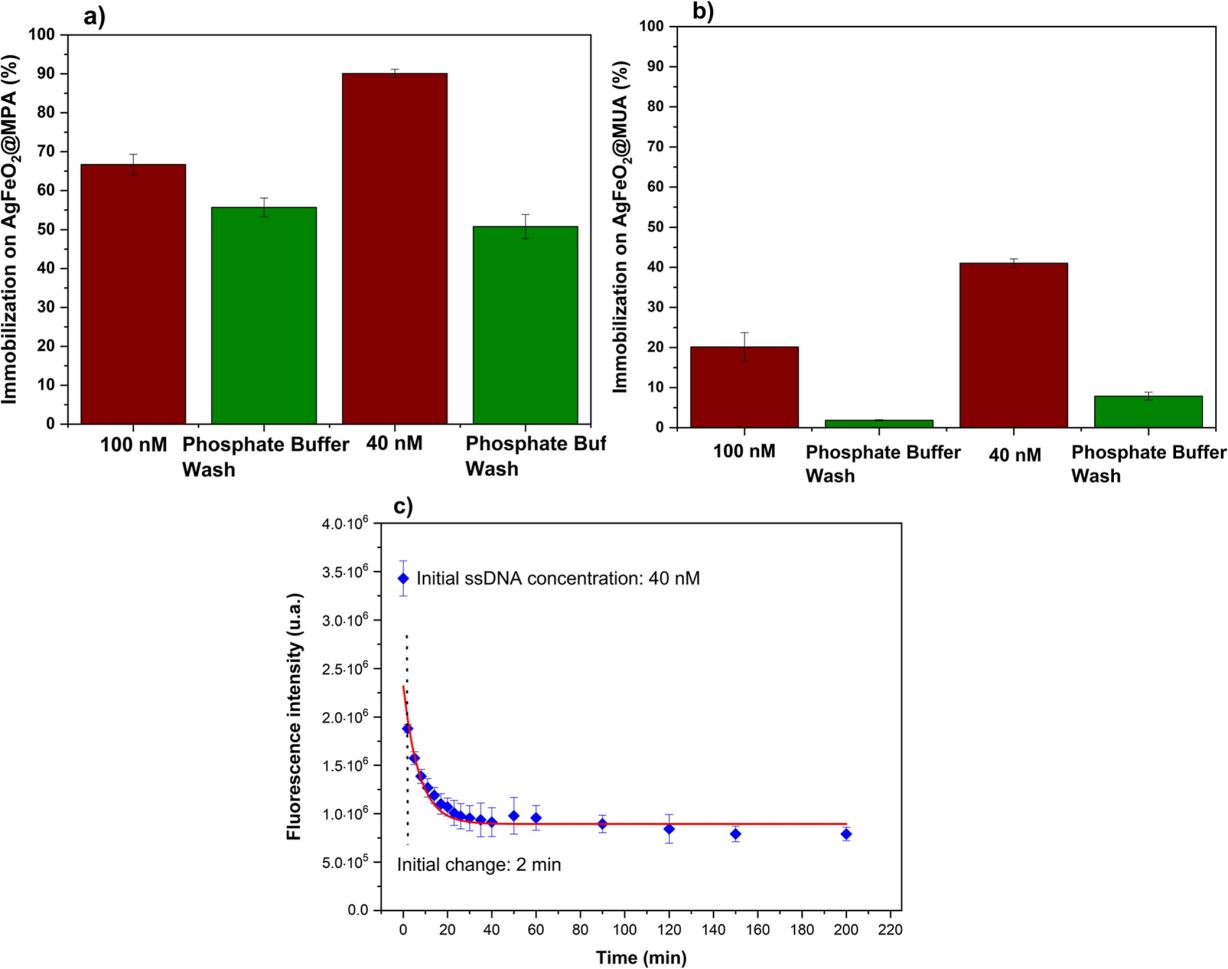
(a) Percentage of FAM-ssDNA immobilization on AgFeO_2_@MPA* nanoparticles. The marsala-colored bars represent the amount of ssDNA immobilized on the nanoparticle surface, while the green-colored bars indicate the amount of ssDNA released after phosphate buffer washing. The difference between these values corresponds to the fraction covalently attached to the surface. (b) Percentage of FAM-ssDNA immobilization on AgFeO_2_@MUA* nanoparticles. (c) Kinetics of FAM-ssDNA immobilization on AgFeO_2_@MPA* nanoparticles.

Figure 3b shows the platform performance when using a longer-chain bifunctional ligand, 11-mercaptoundecanoic acid (11-MUA). The increased chain length is expected to reduce nonspecific ssDNA adsorption and promote more efficient covalent amide bond formation. A comparable immobilization percentage was obtained for AgFeO_2_ functionalized with 11-MUA, indicating overall loading behavior like that of AgFeO_2_@MPA.

Although the longer 11-MUA molecule acts as a more effective “protective” barrier, reducing the likelihood of nonspecific interactions, in both cases, washing with phosphate solution removed most of the weakly attached ssDNA segments.^61^ This test helped determine the optimal ssDNA concentration for saturating the active sites on the particle surface and assess the effect of the longer chain ligand on the sensing platform. Since the same behavior was observed when using the long-chain ligand, we chose to use the shorter 3-MPA ligand for subsequent experiments in constructing the biosensor platform.

#### 4.3.1 Kinetics of Immobilization of FAM-Labeled NH_2_-ssDNA

We allowed 0.5 mg of AgFeO_2_@MPA particles to interact with 1 mL of a 40 nmol·L^–1^ solution of FAM-ssDNA, under gentle agitation at room temperature. In Figure 3c, we show the decrease in fluorescence intensity of the solution as immobilization proceeds, and the ssDNA chains progressively attach to the particle surfaces. As observed, there is a noticeable variation in the intensity within the first 2 min, with saturation beginning to occur after 10 min.

The collected AgFeO_2_@MPA@ssDNA particles were then washed with a 0.1 mol·L^–1^ phosphate solution for 10 min. We observed that although the FAM-ssDNA immobilization process reaches saturation at 40 min, there is no noticeable difference in the amount of loosely bound ssDNA chains released after just 10 min of interaction.

#### 4.3.2 Active Site Blocking Experiments

We performed blocking experiments to minimize nonspecific interactions by adsorbing a large molecule onto the nanoparticle surface, ensuring that CS-DNA hybridizes only with the immobilized ssDNA rather than with the silver–iron oxide particles themselves. For this purpose, 0.5 mg of the AgFeO_2_@MPA/ssDNA complex was incubated with 1 mL of a 0.5 mol L^–1^ BSA solution for 16 h at 5 °C. Figure SI5 presents the results of this experiment, along with an evaluation of Na_2_HPO_4_ as an alternative blocking agent, in which different blocking times were tested and compared with the platform’s response in the absence of any blocker.

### 4.4 Hybridization Procedure: Fluorescence Assessment of FAM-Labeled Complementary NH_2_-ssDNA

We conducted a preliminary study of the hybridization process (second step) to establish the optimal time and concentration for successful hybridization on the platform, using FAM-labeled complementary *Salmonella* spp. ssDNA. Once these parameters were determined, subsequent hybridization tests were carried out using nonlabeled ssDNA immobilization strands.

In the hybridization tests, the AgFeO_2_@MPA@ssDNA complex was placed in contact with the target DNA solution (CS-DNA) and with noncomplementary FAM-labeled DNA (NCS-DNA) to monitor the process via fluorescence. When the AgFeO_2_@MPA@ssDNA particles interact with the complementary target probes, hybridization occurs, forming AgFeO_2_@MPA@dsDNA. Using an external magnetic field, we then remove the AgFeO_2_@MPA@dsDNA complex particles, resulting in a corresponding decrease in fluorescence intensity due to the hybridization event on the particles’ surfaces that were removed from the medium. However, complementary probes may also bind to the AgFeO_2_@MPA@ssDNA particles through weak surface interactions that do not correspond to actual hybridization events. Therefore, the blocking experiment summarized in Figure SI5 provides essential insight into this optimization step.

#### 4.4.1 Fluorescence Assessment of FAM-Labeled Complementary NH_2_-ssDNA: Comparing the Use of MPA and MUA Ligands

Initially, we studied the behavior of AgFeO_2_@MPA/ssDNA and AgFeO_2_@MUA/ssDNA with 5 nmol·L^–1^ of CS-DNA-FAM or NCS-DNA-FAM, diluted in a hybridization buffer. Figure 4a shows the recognition efficiency, estimated according to *f*_recog_ = 100 × (*I*_0_ – *I*_SN_)/*I*_0_, where *I*_0_ and *I*_SN_ are the fluorescence intensities of the initial ssDNA solution and the supernatant collected after removal of the AgFeO_2_@MPA/ssDNA nanoparticles, respectively, during the washing process.

**Figure 4:**
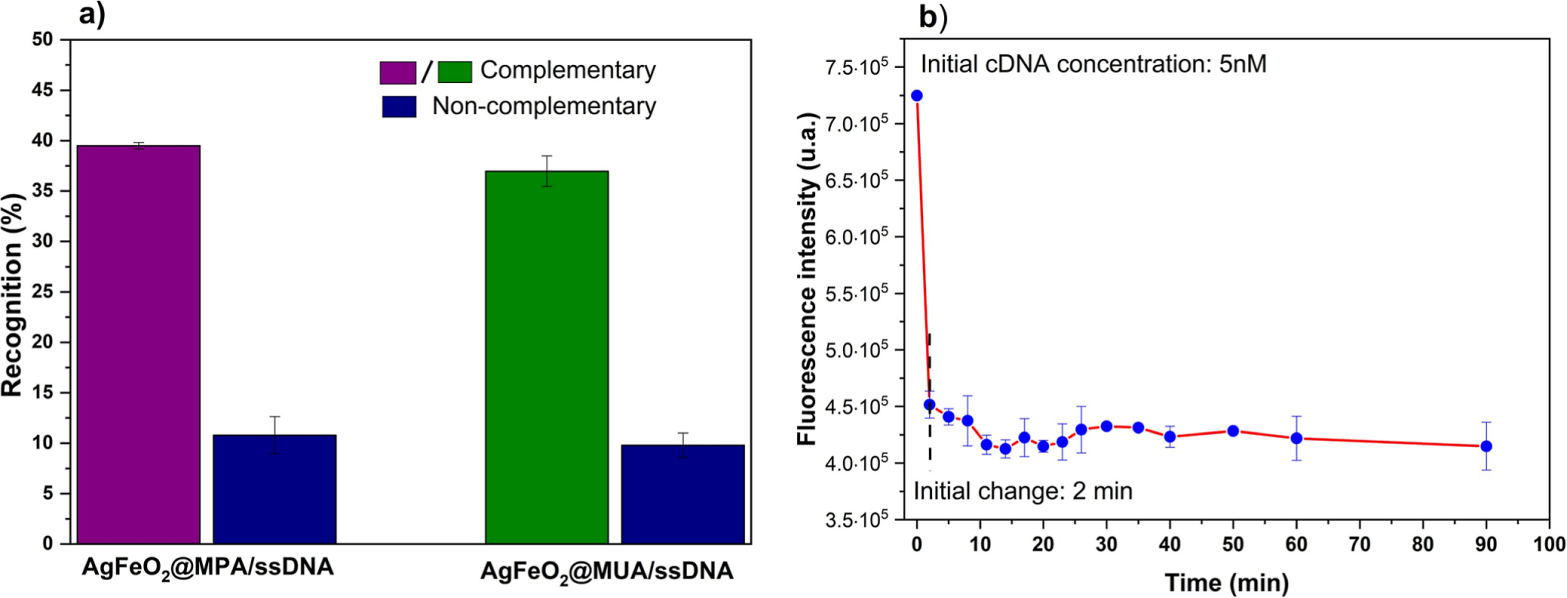
(a) Recognition efficiency of CS-DNA-FAM (complementary, purple rectangle for the MPA system and green rectangle for the MUA system) and NCS-DNA-FAM (noncomplementary, blue rectangles) after the hybridization event on AgFeO_2_@MPA/ssDNA and AgFeO_2_@MUA/ssDNA nanoparticles. (b) Kinetics hybridization of CS-DNA-FAM with AgFeO_2_@MPA/ssDNA nanoparticles.

This recognition percentage is obtained after the washing procedure with 0.1 mol·L^–1^ phosphate solution and corresponds to the specific interaction between AgFeO_2_@MPA/ssDNA and CS-DNA (represented by the purple-colored and green-colored rectangles). For NCS-DNA (blue-colored rectangle), the interactions correspond to weak surface interactions not associated with actual hybridization events.

As observed, in both cases, there is a significant difference between the responses to complementary and noncomplementary DNA probes of the *Salmonella* spp. pathogen, confirming that the proposed platform generates a noticeable positive response when complementary DNA probes are present in the tested solution.

The decrease in fluorescence intensity of the supernatant after removal of the AgFeO_2_@MPA/dsDNA nanoparticles, associated with the hybridization event, was used to calculate the recognition efficiency. The recognition efficiencies were comparable for both systems: 39.50 ± 0.32% for AgFeO_2_@MPA and 36.97 ± 1.52% for AgFeO_2_@MUA. For comparison, a decrease of approximately 10%—attributed to nonspecific interactions—was observed when noncomplementary probes were used at a concentration of 5 nmol L^–1^, with 1 mL of either CS-DNA or NCS-DNA employed in all experiments.

We also used a washing process with 0.1 mmol·L^–1^ phosphate buffer to remove any potential weak interactions between the AgFeO_2_@MPA/ssDNA platform and the DNA strand. The fact that the largest response was observed with complementary DNA probes confirms that, under the present protocol, both systems are effective for biosensing applications.

Figure SI6 shows the results of the hybridization experiments performed using different hybridization buffers. For the AgFeO_2_@MPA/ssDNA platform, the best performance was obtained with the 6× SSC (saline–sodium citrate) buffer, a formulation commonly used in biotechnology and biosensor fabrication.^62^ After confirming the sensor’s effectiveness, the kinetics of the hybridization process with complementary sequences were investigated. Figure 4b shows the variation in fluorescence intensity of the supernatant, measured after removal of the AgFeO_2_@MPA/dsDNA nanoparticles, as a function of exposure time. A pronounced decrease in fluorescence intensity was observed during the first few minutes of interaction, followed by gradual saturation of the hybridization process for the complementary FAM-ssDNA over longer periods.

### 4.5 Analytical Sensitivity of *Salmonella* spp. DNA Detection

To determine the sensing range of the proposed platform, which is based on the use of AgFeO_2_@MPA/ssDNA particles for detecting CS-DNA sequences of *Salmonella* spp., as well as to estimate the corresponding detection limit, we conducted a detailed series of experiments using varying concentrations of the CS-DNA-FAM probe in the range of 0.5–10 nmol·L^–1^. In Figure 5a, we present the corresponding change in recognition percentage as a function of CS-DNA-FAM probe concentration extracted from the fluorescence plot curve (inserted in Figure 5a). As shown in Figure 5a, a linear relationship is observed within the concentration range used, with the recognition percentage being inversely proportional to concentration.

**Figure 5:**
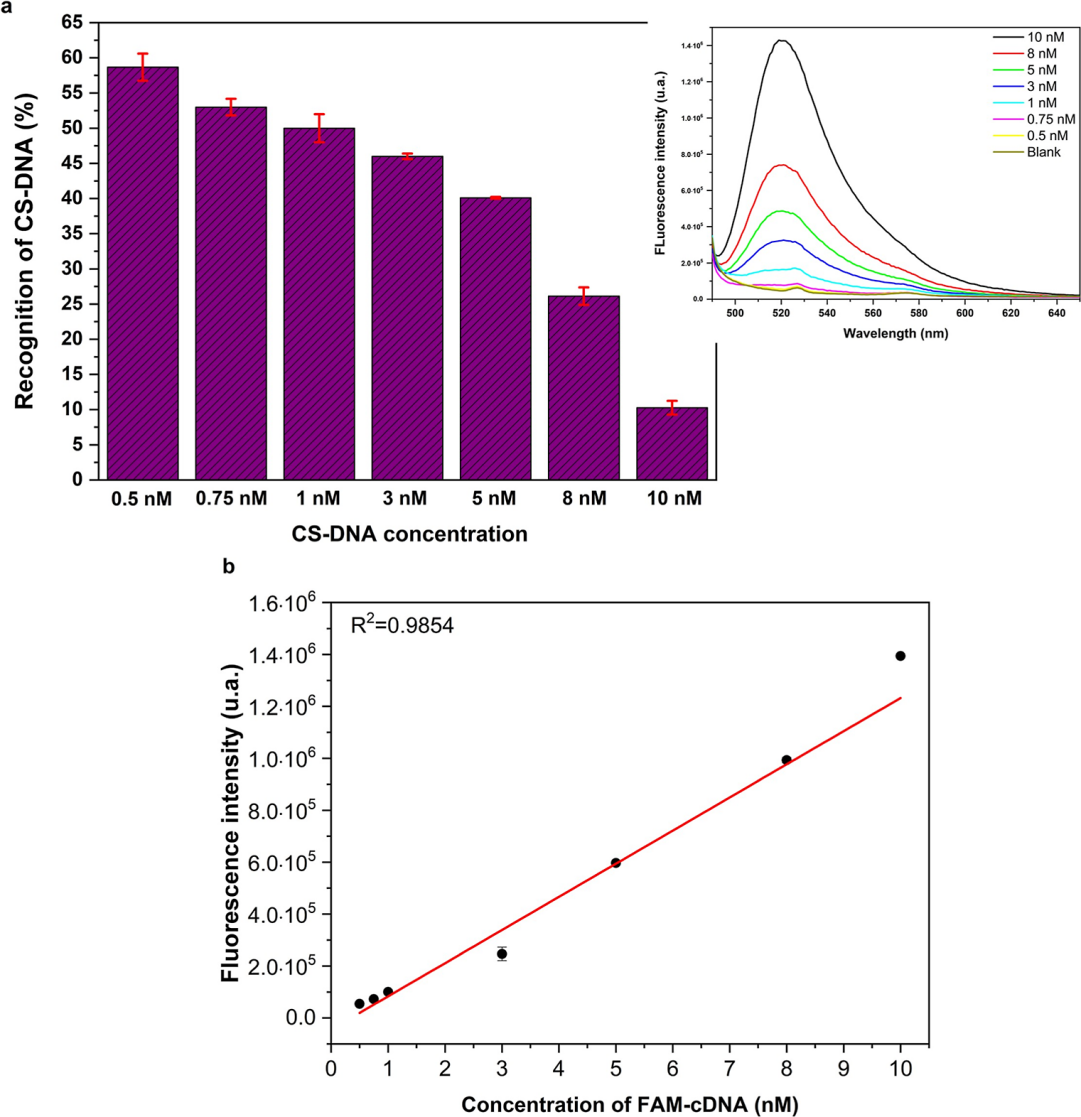
(a) Analytical sensitivity of the AgFeO_2_@MPA/ssDNA platform for recognizing CS-DNA-FAM after hybridization. (b) Linear response of the AgFeO_2_@MPA/ssDNA platform.

At lower concentrations of CS-DNA-FAM, higher recognition percentages are observed. For instance, at 0.5 nmol·L^–1^ of CS-DNA-FAM, the AgFeO_2_@MPA/ssDNA biosensor (0.5 mg) exhibits a recognition percentage of approximately 60% (58.74 ± 1.93). In contrast, at higher concentrations, such as 8 nmol·L^–1^ of CS-DNA-FAM, the recognition percentage decreases to approximately 27% (26.13 ± 1.25). Additionally, when 10 nmol·L^–1^ of CS-DNA-FAM is used, the biosensor achieves a recognition percentage of about 10% (10.26 ± 0.98). This decline is attributed to the saturation of active sites on the particle surfaces; at this concentration, the recognition percentage becomes comparable to that observed for NCS-DNA, suggesting weak, nonspecific interactions.

In this context, the optimal concentration range for the proposed biosensor is below 10 nmol·L^–1^. This is related to the active surface of the biosensor particles; in other words, the ssDNA immobilized on the particles facilitates the recognition of lower concentrations of CS-DNA. In an ideal system, each immobilized DNA strand on the surface would hybridize with one CS-DNA strand. However, in a real system, DNA strands can accumulate around the particle, preventing efficient hybridization. In this scenario, the approach of the CS-DNA strand is sterically hindered by the immobilized DNA strands, leading to the behavior shown in Figure 5a. For 0.5 mg of AgFeO_2_@MPA/ssDNA, a lower concentration of CS-DNA is needed to obtain a robust result. When a 50 nmol·L^–1^ CS-DNA-FAM solution was tested, the percentage of recognition (PoR) remained at the same level (10%) as that observed for a 10 nmol·L^–1^ CS-DNA-FAM solution, indicating that saturation of the active sites on the AgFeO_2_@MPA/ssDNA particles had already begun to occur at this lower concentration.

Figure 5b displays the pristine fluorescence data—that is, the raw intensity values obtained directly from the instrument—plotted as a function of CS-DNA-FAM concentration. A linear fluorescence response was observed after interaction with the AgFeO_2_@MPA/ssDNA biosensor, confirming the platform’s quantitative behavior.

The increase in fluorescence intensity (I) with the concentration of the target FAM-cDNA solution [x] in the range of 0.5 to 10 nmol·L^–1^ can be fitted to a linear equation (*R* ^2^ = 0.985) given by *I* = 12764.7 × *x* – 44260.9. Using this as a calibration curve and applying the relation 3σ/S,^63^ where σ is the standard deviation of 10 samples of the blank, and S is the slope of the calibration curve, we estimated the limit of detection (LoD) of the proposed sensing platform to be 73 pmol·L^–1^ (0.0727 nmol·L^–1^).

The calculated limit of detection (LoD) was obtained using purified DNA sequences, demonstrating excellent sensitivity and confirming the high intrinsic responsiveness of the AgFeO_2_-based platform to target nucleic acids. To establish its correlation with *Salmonella* cells, it is necessary to estimate the number of gene copies per cell and convert this to a concentration in cells per milliliter.

For this

$$\frac{molecules}{mL}=C\times N_{A}=\left(\frac{7{3\times 10}^{-12}\;mol}{L}\right)\times \left(6.022\times \frac{10^{23}\;molecules}{mol}\right)==4.40\times 10^{13}\frac{molecules}{L}\times \left(\frac{1\;L}{1000\;mL}\right)=4.40\times \frac{10^{10}\;molecules}{mL}$$

Assuming that in one cell in the stationary phase, has one copy of the target gene

$$\frac{Cell}{mL}=\left(\frac{molecules}{mL}\right)÷\left(N_{copyofgen}\right)=\left(4.40\times 10^{10}\;\frac{molecules}{mL}\right)÷\left(1\;copy\;of\;gene\;per\;cell\right)=4.40\times 10^{10}\;cell/mL$$

For the AgFeO_2_@MPA/ssDNA biosensor, the limit of detection (LoD) of 73 pmol L^–1^ within the analytical range corresponds to approximately 4.40 × 10^10^ cells mL^–1^, or about 4.4 × 10^7^ DNA copies μL^–1^. Under direct detection conditions—without any DNA amplification—this value represents a similar number of bacterial cells per microliter. The genome of *Salmonella enterica* contains approximately 4.8–5.0 × 10^6^ base pairs, equivalent to roughly 5 femtograms (fg) of DNA per cell.

Although the present study establishes the analytical performance of the AgFeO_2_@MPA/ssDNA platform using purified synthetic oligonucleotides under controlled conditions, translation to real-world samples will require additional preanalytical processing. Such steps may include filtration, centrifugation, DNA extraction and purification, and, when necessary, amplification strategies such as PCR or isothermal methods. These aspects are beyond the scope of the present proof-of-concept study and constitute the focus of ongoing and future work aimed at validating sensor performance in complex biological and environmental matrices.

### 4.6 Comparative Evaluation of the AgFeO_2_@MPA/ssDNA Biosensor and Related DNA-Sensing Platforms

Table 2 compares the performance of the AgFeO_2_@MPA/ssDNA platform with other biosensing systems used for quantifying *Salmonella* spp., considering both the detection method (fluorescent or electrochemical) and the length of the purified DNA sequences employed.

**Table 2:** Performance of the AgFeO_2_@MPA/ssDNA Platform for ssDNA Identification Compared to Structures Reported in the Literature^a^

structure	length of oligonucleotides	analyzed range	sensor response	detection limit	ref
NaYF_4_:Yb,Er@SiO_2_/ GO nanoparticles	*I*: 21 b	0.1–400 nM	fluorescence	100 pM	^64^
	*C*: 31 b				
carbon nanoparticles	-	33–300 nM	fluorescence	-	^65^
carboxylic Carbon Quantum Dots	*I*: 26 b	0.4–400 nM	fluorescence	45,6 nM	^66^
	*C*: 26 b			17.4 nM	
polydopamine nanospheres	*I*: 26 b	5–30 nM	fluorescence	5 nM	^67^
	*C*: 23 b				
CeO_2_ Nanorods	*I*: 20 b	10^–14^–10^–7^ M	electrochemical (EIS)	10^–15^ M	^68^
	*C*: 20 b				
nanoporous glassy carbon electrode	*I*: 21 b	10 × 10^–12^–400 × 10^–12^ M	electrochemical (DPV)	2.1 × 10^–12^ M	^69^
	*C*: 21 b				
ITO electrodes	-	5–250 nM	electrochemical	2.4 nM	^70^
CeO_2_–NR@Ppy nanocomposite	-	0.01–0.4 nM	electrochemical	0.084 nM	^71^
**AgFeO**_**2**_ **@MPA particles**	*I*: 26 b	0.5–10 nM	fluorescence	0.073 nM (73 pM)	**this work**
	*C*: 26 b				

a I: Immobilization sequence. C: Complementary sequences. b: bases.

For fluorescence-based methods, the limit of detection (LoD) is typically in the nanomolar range, employing various nanostructures and materials. Electrochemical detection, on the other hand, generally achieves a lower LoD than fluorescence methods. It is important to note that the present study introduced approaches using covalent immobilization of ssDNA on inorganic magnetic particles and tested complementary DNA concentrations lower than those listed in Table 2. In this context, the proposed biosensor demonstrates excellent sensitivity and selectivity, with the added advantage of exploiting the magnetic properties of the AgFeO_2_@MPA/dsDNA complex to enable its efficient removal from the analysis medium. The high sensitivity and low detection limit can be attributed to several combined factors, including the nanostructured surface chemistry of the AgFeO_2_ nanoparticles, the optimized immobilization and hybridization conditions, and the carefully controlled washing and purification steps. Overall, the biosensor exhibits performance comparable to or better than that of recently reported platforms for biomolecule detection.

### 4.7 Selectivity of the AgFeO_2_@MPA/ssDNA Platform for the Identification of *Salmonella* spp. ssDNA Probes

Selectivity is a crucial parameter in evaluating a biosensor, as it reflects its ability to distinguish truly complementary sequences from similar ones. Figure 6 compares the percentage of recognition (PoR) obtained when 0.5 mg of the AgFeO_2_@MPA/ssDNA complex was exposed to 1 mL of complementary (CS) and partially mismatched DNA sequences (two base mismatches, as listed in Table 1) at a fixed concentration of 5 nmol L^–1^. The experiments were performed following the same procedure described for hybridization (Section 2.4.1). The maximum PoR (40.1 ± 0.38%) was observed for the complementary ssDNA, consistent with effective hybridization, whereas the minimum value (8.08 ± 4.33%) was obtained for the fully noncomplementary sequences, reflecting only nonspecific adsorption.

**Figure 6:**
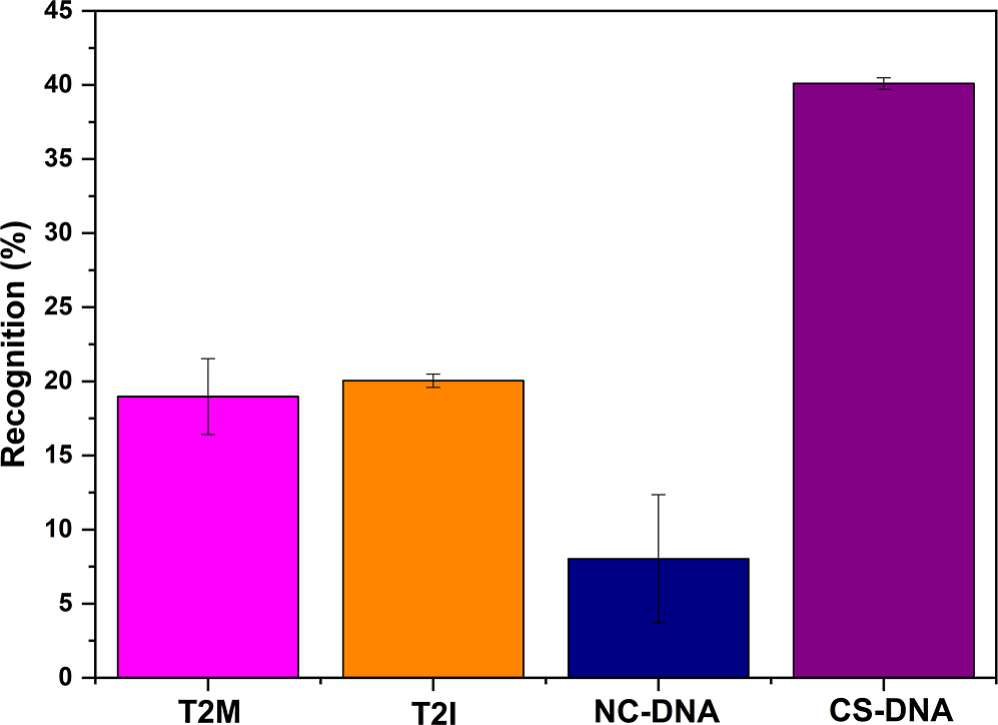
Selectivity of the AgFeO_2_@MPA/ssDNA platform toward different ssDNA sequences: complementary (CS-DNA, purple rectangle), noncomplementary (NCS-DNA, blue rectangle), two-base mismatch in the middle (T2M, pink rectangle), and two-base mismatch at the 5′ end (T2I, orange rectangle).

These PoR values should be compared to the results of 18.85 ± 2.57% and 19.98 ± 0.45%, obtained when sequences with two mismatched bases in the middle (T2M) or at the beginning (T2I) of the ssDNA strand were used, respectively. Thus, the AgFeO_2_@MPA/ssDNA biosensor demonstrated high selectivity for *Salmonella* spp. DNA chains, as even sequences with two mismatched bases were distinguishable. The PoR for CS-DNA was significantly higher 40.1 ± 0.38%, indicating the strong selectivity and robust performance of the AgFeO_2_@MPA/ssDNA platform.

Together, the blocking-agent optimization, hybridization buffer selection, and mismatch discrimination experiments demonstrate that the observed fluorescence responses originate predominantly from specific hybridization events rather than nonspecific adsorption. These results indicate effective suppression of false-positive signals under the controlled conditions employed in this proof-of-concept study.

## 5 Detection of *Salmonella* spp. ssDNA Hybridization Using SYBR Green: On–Off Fluorescent Sensor

To elucidate the mechanism using SYBr-green (SG) as a response to the recognition of CS-DNA of *Salmonella* spp., the hybridization experiment was conducted as described in Section 2.4.1. For this experiment, CS-DNA and NCS-DNA without FAM labeling were used. Since SG is a dye that binds only to dsDNA when a hybridization event occurs, its fluorescence indicates the formation of double-stranded DNA.

In this context, 0.5 mg of AgFeO_2_@MPA/ssDNA particles were exposed to 1 mL of CS-DNA or NCS-DNA at varying concentrations and then washed with a 0.1 mmol·L^–1^ phosphate buffer. The AgFeO_2_@MPA/dsDNA particles were then placed in contact with 1 mL of SYBR Green (SG) diluted to 1× in nuclease-free ultrapure water and gently shaken. Afterward, the AgFeO_2_@MPA/dsDNA particles were deposited on a glass substrate, and the fluorescence of this solid material was analyzed using a Zeiss Axio Imager 2 fluorescence microscope equipped with an Axiocam 503c_s256 camera.

The corresponding fluorescence images are presented in Figure 7. To identify the hybridization event, the 0.5 mg AgFeO_2_@MPA/dsDNA platform was systematically compared with the same platform exposed to 1 mL of NCS-DNA at a fixed concentration of 5 nmol L^–1^.

**Figure 7:**
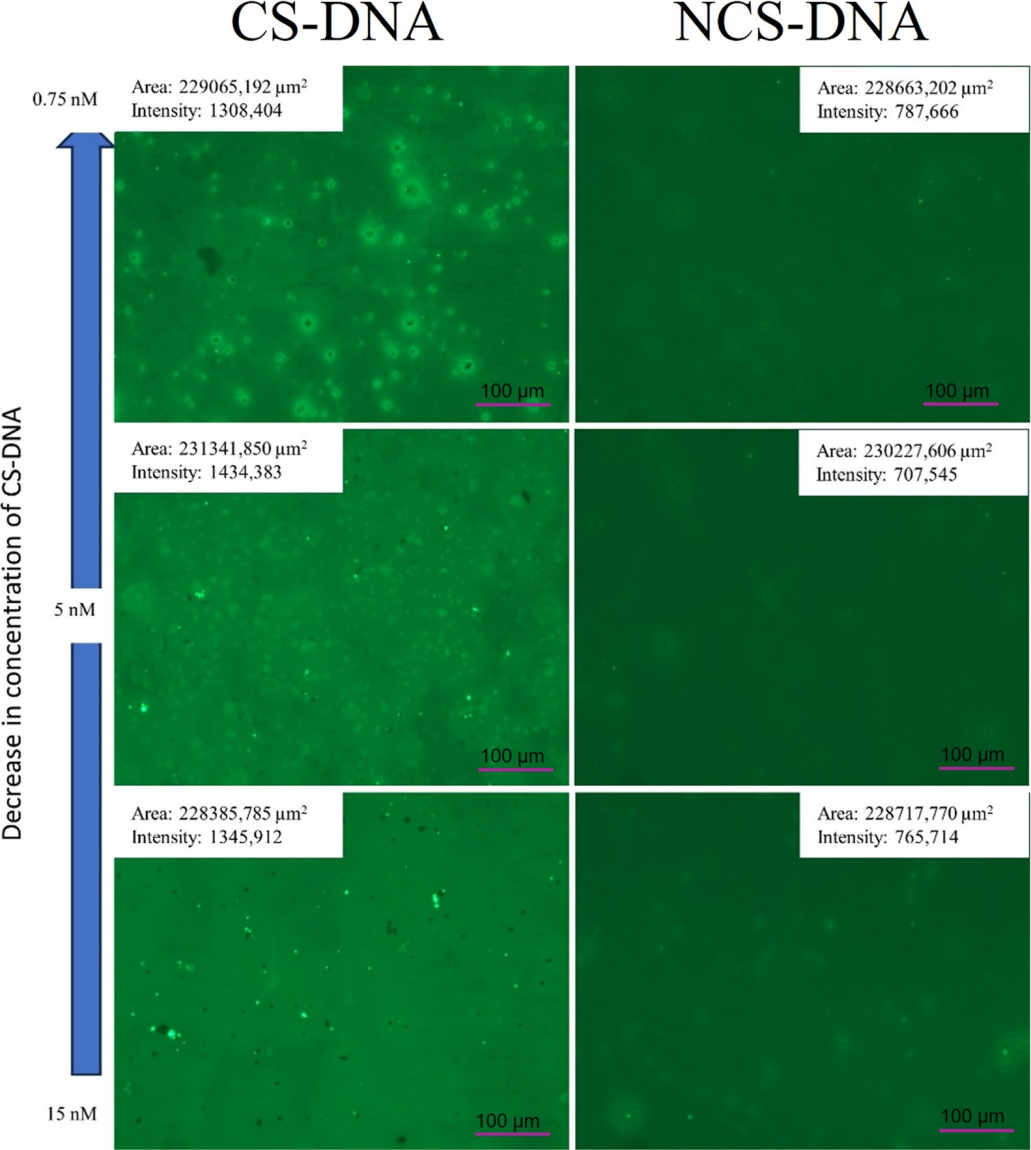
Identification of the hybridization event on the AgFeO_2_@MPA/ssDNA platform using SYBR Green dye for detecting double-stranded DNA (dsDNA) of *Salmonella* spp. The CS-DNA column corresponds to the complementary sequence hybridized with the immobilized ssDNA on AgFeO_2_@MPA/ssDNA nanoparticles, whereas the NCS-DNA column corresponds to the noncomplementary sequence.

To ensure accurate comparisons of the images, parameters such as light exposure and analysis area were controlled. For concentrations of 0.75 nmol·L^–1^ and 5 nmol·L^–1^ of CS-DNA, positive recognition becomes evident, with a brightness increase of almost 50% compared to the negative signal (NCS). For positive recognition, intensity values of 1308.40, 1434.38, and 1345.91 were obtained for 0.75 nmol·L^–1^, 5 nmol·L^–1^, and 15 nmol·L^–1^ CS-DNA, respectively. In contrast, the negative signal (NCS) showed intensity values of around 750 counts per second (CPS). This indicates effective hybridization on the platform. The lack of further increase in intensity at 15 nmol·L^–1^ suggests that the platform has reached saturation of its active sites.

Consequently, with this result and the evident difference between the positive signal (fluorescence intensity of 1308.40 CPS) and the negative signal (fluorescence intensity of 750 CPS), it can be deduced that the AgFeO_2_@MPA/ssDNA biosensor can also function as an on–off sensor. The results are robust and reproducible, supporting the conclusion that this biosensor is effective for nucleic acid detection and has potential applications in the fields of biosensing and biotechnology.

## 6 Conclusions

In this study, we developed a biosensing platform based on functionalized silver ferrite (AgFeO_2_) nanoparticles for detecting *Salmonella* spp. DNA sequences. The bifunctional ligand 3-mercaptopropionic acid (3-MPA) enabled robust covalent immobilization of ssDNA, enhancing both sensitivity and selectivity. The resulting AgFeO_2_@MPA/ssDNA biosensor effectively recognized complementary DNA strands, producing a pronounced fluorescence contrast relative to noncomplementary sequences and confirming the platform’s robustness and reliability.

The kinetic study of immobilization and hybridization revealed rapid response times, with signal saturation occurring in under 40 min. The analytical limit of detection (LoD) was estimated at 73 pmol L^–1^ (0.073 nmol L^–1^), corresponding to roughly 4.4 × 10^7^ DNA copies μL^–1^. These results show that the platform is highly competitive with other *Salmonella* detection systems, demonstrating excellent performance even at low target DNA concentrations using purified sequences. Moreover, the biosensor’s ability to discriminate sequences containing base mismatches, as demonstrated in the selectivity assays, underscores its potential for precise molecular detection in complex biological samples.

The use of SYBR Green dye for fluorescence detection, combined with the platform’s magnetic properties that enable easy removal of hybridized AgFeO_2_@MPA/dsDNA nanoparticles, adds significant practicality, making the biosensor suitable for on–off detection schemes. This versatility underscores its potential for broad application in rapid and real-time diagnostic settings.

Overall, this work makes a significant contribution to biosensor development by introducing a magnetic, fluorescence-based platform that combines high sensitivity, selectivity, and operational simplicity. The magnetic properties, facile functionalization, and robust DNA-recognition performance highlight the AgFeO_2_@MPA/ssDNA system as a promising tool for applications in biotechnology, medical diagnostics, and food safety, with strong potential for integration into next-generation biosensing technologies.

Although this proof-of-concept study validated the physicochemical and functional performance of the sensing mechanism under controlled conditions using synthetic ssDNA, future work will focus on extending the platform to real biological samples containing longer dsDNA and complex microbial environments. This will require coupling the sensing protocol to preanalytical steps such as filtration, centrifugation, DNA extraction and purification, denaturation, and amplification strategies (e.g., PCR or LAMP). Future studies may also include SEM imaging and DLS measurements in different media to further evaluate particle morphology and colloidal stability under real-matrix conditions.

## Supplementary Material



## Notes

Figure SI.1. Schematic Representation of AgFeO_2_ Particle Synthesis and Functionalization Figure SI.2. FTIR Spectra of AgFeO_2_ and AgFeO_2_@MPA Particles Figure SI.3. STEM Images of AgFeO_2_ Particles Figure SI.4. Magnetic Behavior of AgFeO_2_ Particles Figure SI.5. Evaluation of BSA and Na_2_HPO_4_ as blocking agents of active sites on the AgFeO_2_@MPA/ssDNA platform, compared with the unblocked condition. Figure SI.6. Hybridization Experiment with Different Hybridization Buffers (PDF)
