# Supplementary material for: Functionalized AgFeO2 Particles as Fluorescent Platforms for the Detection of Salmonella spp
Source: ACS Omega. 2026 Mar 7;11(11):17172–85. doi: 10.1021/acsomega.5c07267 (PMC13019410; doi:10.1021/acsomega.5c07267)
Supplement: Supplementary file 1 [file ao5c07267_si_001.pdf]

Supporting Information

Functionalized $AgFeO_2$ Particles as Fluorescent Platforms for the Detection of *Salmonella* spp.

Lizeth C. Mojica-Sánchez[a], Gabriela P. Ratkovski[a], Bruna G. Maciel[b], Winnie Q. Brandão[b], Romário J. da Silva[a], Celso P. de Melo[a,b]

[a]*Departamento de Física, Universidade Federal de Pernambuco, 50670-901 Recife, PE, Brazil*
[b]*Pós-Graduação em Ciência de Materiais, Universidade Federal de Pernambuco, 50670-901 Recife, PE, Brazil*

1. Schematic Representation of $AgFeO_2$ Particle Synthesis and Functionalization

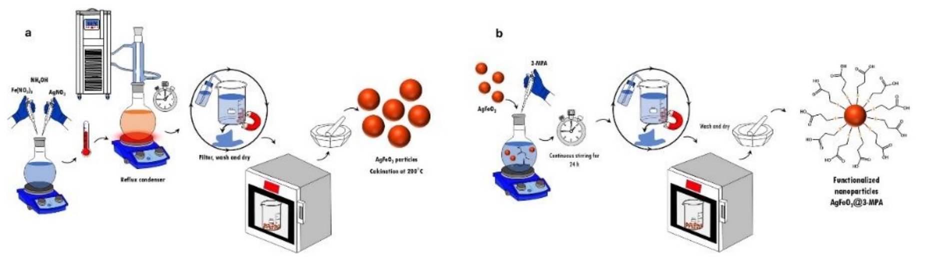

Figure SI.1. (a) Schematic representation of the synthesis process for silver ferrite ($AgFeO_2$) particles. (b) Functionalization procedure using the 3-mercaptopropionic acid (3-MPA) molecule.

2. FTIR Spectra of $AgFeO_2$ and $AgFeO_2$@MPA Particles

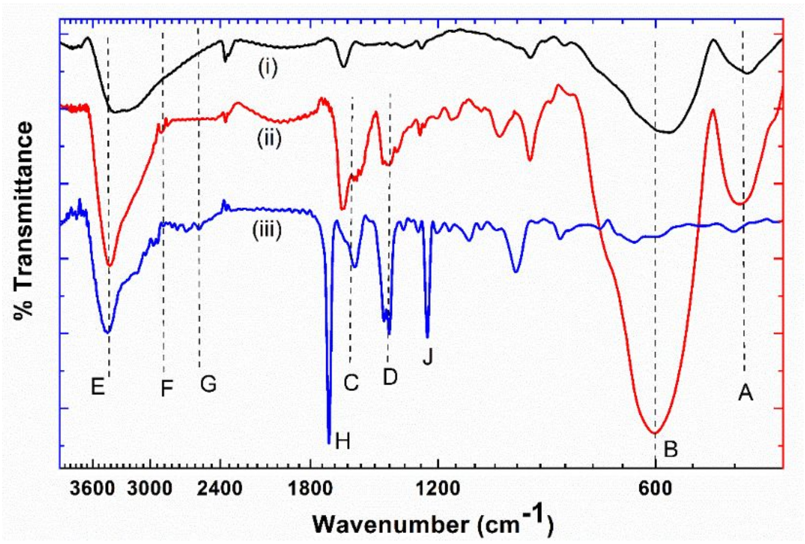

Figure SI.2. FTIR spectra of unmodified $AgFeO_2$ particles (curve i), $AgFeO_2$ particles functionalized with 3-MPA ($AgFeO_2$@MPA, curve ii), and the 3-MPA ligand (curve iii).

3. STEM Images of $AgFeO_2$ Particles

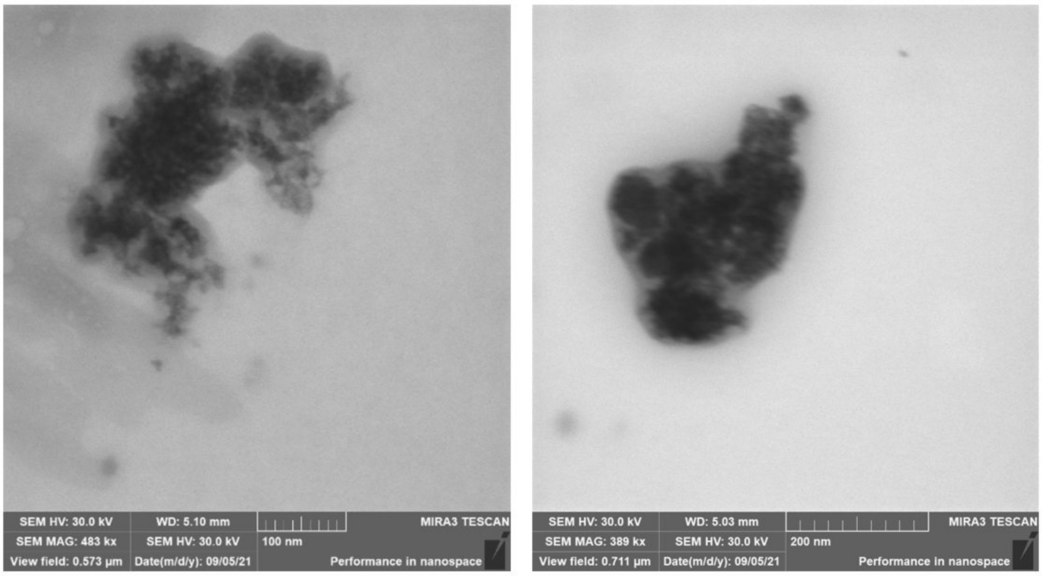

Figure SI.3. STEM images of $AgFeO_2$ nanoparticles, highlighting particle agglomeration, which is likely caused by magnetic interactions.

4. Magnetic Behavior of $AgFeO_2$ Particles

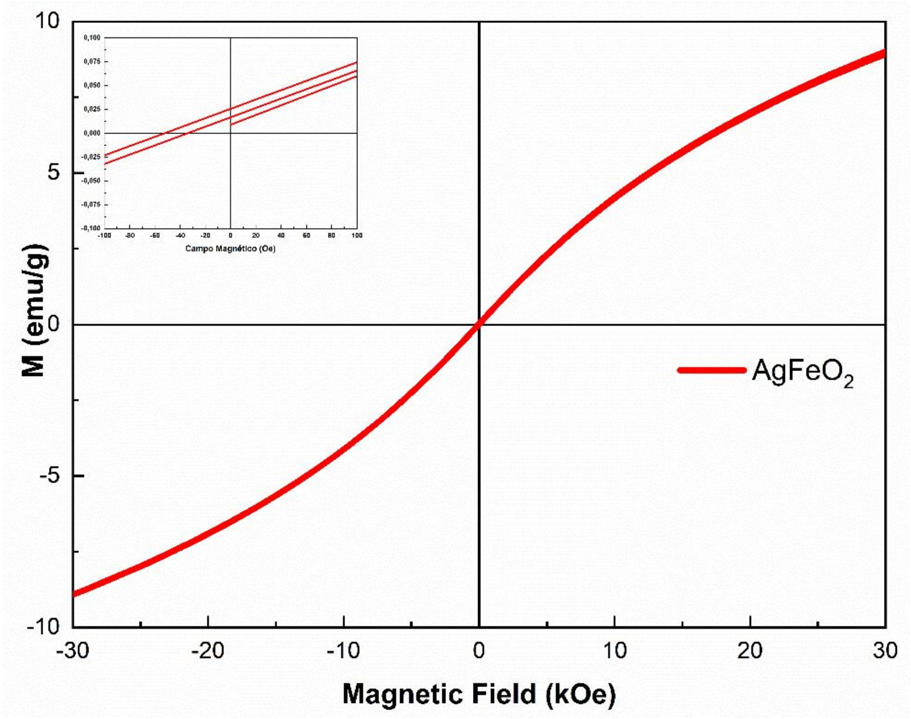

Figure SI.4. Room-temperature hysteresis curve of $AgFeO_2$ particles, indicating a soft ferromagnetic behavior.

5. Use of BSA and Na_2HPO_4 as Blocking Agents of Active Sites in $AgFeO_2$@MPA/ssDNA

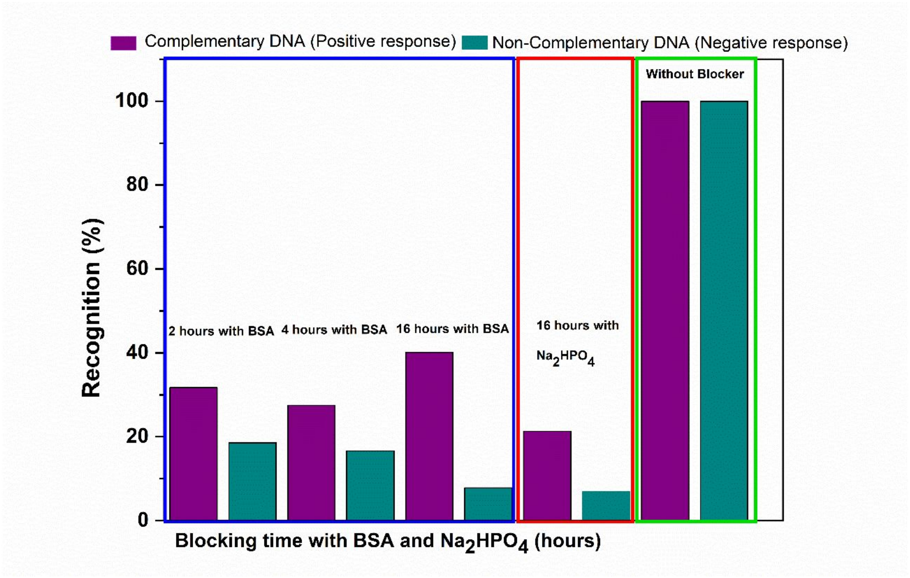

Figure SI.5. Study of blocking time using BSA and Na_2HPO_4 as blocking agents to minimize nonspecific interactions, compared with systems lacking any blocker. The purple bars represent the amount of complementary DNA (CS-DNA) bound to the nanoparticle surface (specific response), while the green bars indicate the amount of noncomplementary DNA (NCS-DNA) bound via nonspecific interactions. For BSA, blocking times of 2, 4, and 16 h were evaluated to determine the optimal duration yielding the greatest difference between CS-DNA and NCS-DNA signals. Na_2HPO_4 was tested at 16 h and exhibited lower DNA recognition levels; therefore, it was selected as the blocking agent for subsequent experiments. In the samples without a blocker, no distinction between positive and negative responses was observed, indicating that both CS-DNA and NCS-DNA were adsorbed through nonspecific physical interactions, making sequence-specific detection unfeasible.

6. Hybridization Experiment with Different Hybridization Buffers

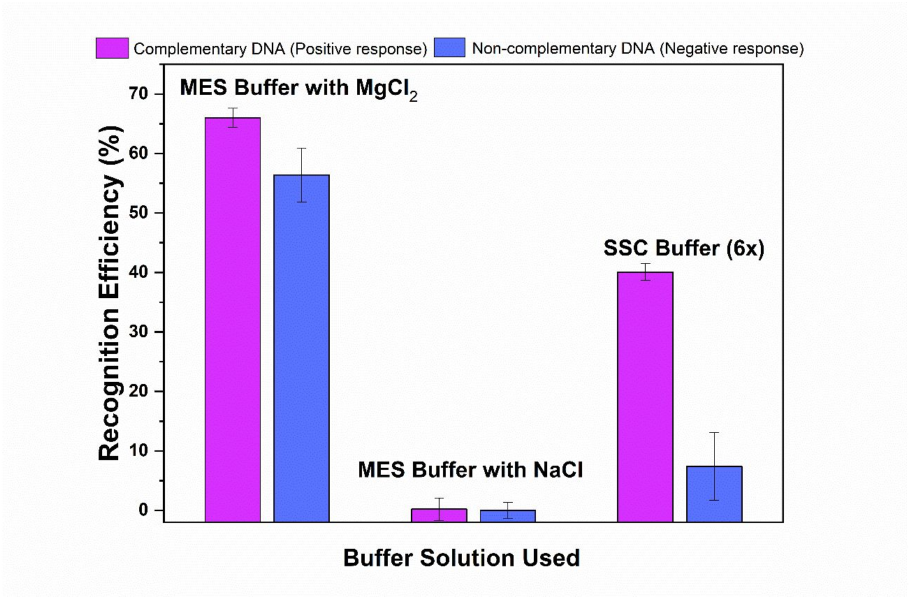

Figure SI.6. Hybridization of CS-DNA-FAM on $AgFeO_2$@MPA*/ssDNA using different hybridization buffers. The purple bars represent the amount of complementary DNA (CS-DNA) on the particle surfaces (positive response), while the blue bars represent the amount of non-complementary DNA (NCS-DNA) (nonspecific interactions). Three hybridization buffers were tested to identify the one that provided the largest difference between CS-DNA and NCS-DNA. For the MES:$MgCl_2$ buffer, nonspecific interactions with NCS-DNA were observed. The MES:NaCl buffer did not allow the hybridization event to occur. In contrast, the SSC 6x buffer provided the greatest difference between CS-DNA and NCS-DNA, enabling the hybridization event. Therefore, SSC 6x was selected as the optimal hybridization buffer for all experiments.
